# Covid‐19 and risk of acute pancreatitis: What beyond

**DOI:** 10.1111/jcmm.18082

**Published:** 2023-12-15

**Authors:** Hayder M. Al‐kuraishy, Ali I. Al‐Gareeb, Athanasios Alexiou, Marios Papadakis, Mostafa M. Bahaa, Gaber El‐Saber Batiha

**Affiliations:** ^1^ Department of Clinical pharmacology and Medicine, College of Medicine Mustansiriyah University Baghdad Iraq; ^2^ Department of Science and Engineering Novel Global Community Educational Foundation New South Wales Australia; ^3^ AFNP Med Wien Austria; ^4^ Department of Surgery II, University Hospital Witten‐Herdecke, Heusnerstrasse 40 University of Witten‐Herdecke Wuppertal Germany; ^5^ Pharmacy Practice Department, Faculty of Pharmacy Horus University New Damietta Egypt; ^6^ Department of Pharmacology and Therapeutics, Faculty of Veterinary Medicine Damanhour University Damanhour Egypt

Dear editor

1

We read with great interest the article ‘Covid‐19 and acute pancreatitis: examining the causality’ published in 2021 by de‐Madaria and Capurso in Nature Review Gastroenterology and Hepatology.^1^ The article highlights the great importance of acute pancreatitis (AP) in coronavirus disease‐2019 (Covid‐19) which might be as high as 24%. As well, they elaborated the possible role of angiotensin‐converting enzyme 2 (ACE2) and transmembrane protein serine 2 (TMPRSS2) in the pathogenesis of severe acute respiratory syndrome coronavirus 2 (SARS‐CoV‐2)‐induced AP. The authors in an elegant way described that SARS‐CoV‐2 can spread from the duodenal epithelium via the pancreatic duct to the acini and islet cells.

We would like to elaborate and discuss other possible and putative mechanisms that might be concerned in the etiopathogenesis of AP in Covid‐19. First, we would find the link between SARS‐CoV‐2 infection and the risk of AP. A prospective multi‐centre study involved 1777 patients with AP, 149 patients (8.3%) had Covid‐19, and they had a great risk for development of acute respiratory distress syndrome (ARDS) and mortality.^2^, ^3^, ^4^ Thus, patients with AP with Covid‐19 are at high risk for the development of severe AP and correlated with long hospital stay, poor clinical outcomes and 30‐day mortality.^2^ The mortality and severity of AP are increased in Covid‐19 patients and are linked with high mortality due to the development of hyperinflammation.^5^

Other mechanisms which could engage with the development of AP in severe SARS‐CoV‐2 infection are the expression of CD147.^6^ It has been shown that CD147 is regarded as a putative receptor for SARS‐CoV‐2 and its variants. Inhibition of CD147 by anti‐CD147 meplazeumab can reduce entry of SARS‐CoV‐2 and its variants.^6^ CD147 is highly expressed in pancreatic islet cells that increases risk for the interaction with SARS‐CoV‐2 and subsequent development of AP.^7^ Notably, the expression of CD147 in the pancreatic islet cells is higher in diabetic patients that may explain susceptibility of diabetic patients for development of severe Covid‐19.^7^ Activation of CD147 during SARS‐CoV‐2 infection induces stimulation of extracellular cyclophilin A (CyPA) which promote inflammation through activation of different signalling pathways including nuclear factor kappa‐light‐chain‐enhancer of activated B cells (NF‐κB), extracellular signal‐regulated protein kinases 1 and 2 (ERK1/2) and mitogen‐activated protein kinase (MAPK).^6^, ^7^ As well, CD147/CyPA axis promotes chemotaxis of inflammatory cells in the pancreatic tissues. These observations suggest that activation of CD147/CyPA in severe SARS‐CoV‐2 infection may lead to development of cytokine storm and AP.^6^, ^7^

Furthermore, progress of cytokine storm in severe Covid‐19 may cause development of AP.^8^ Hegyi et al. observed that alterations of cytokine pattern are similar in both AP and Covid‐19. Hypercytokinemia in AP with Covid‐19 could be related to SARS‐CoV‐2‐induced lipotoxicity.^8^ Notably, autopsy data proposes that the incidence of AP in Covid‐19 may be higher as compared with diagnosed clinically.^8^ SARS‐CoV‐2 not only targets the pancreas, but it affects adipocytes causing lipolysis and lipotoxicity.

Indeed, severe SARS‐CoV‐2 infection may associate with propagation of gut dysbiosis which may lead to the development of cytokine storm.^9^ Into the bargain, Zhu et al. found that gut dysbiosis was linked with severity of AP in both human and mice.^10^ Further, preserving of gut flora may decrease the systemic inflammatory disorders in AP. However, the potential link between gut dysbiosis and development of AP is not yet completely understood. Therefore, SARS‐CoV‐2 infection‐induced gut dysbiosis could be a potential mechanism of AP in severely affected Covid‐19 patients. Restoration of gut homeostasis by using probiotic therapy may reduce risk for propagation of AP in Covid‐19.

Moreover, neuropilin‐1 which is membrane bound co‐receptor for vascular endothelial growth factor (VEGF) and semaphorin, plays a critical role in angiogenesis, cell survival, invasion, and migration. Neuropilin‐1 is highly expressed in pancreatic tissue.^11^ SARS‐CoV‐2 exploits neuropilin‐1 as an entry point into the affected cells. Neuropilin‐1 overexpression is linked with development of AP by activating release of pro‐inflammatory cytokines.^12^ Targeting of pancreatic neuropilin‐1 receptors by specific inhibitors can reduce AP‐induced splenic injury and progression of systemic inflammation.^12^

Taken together, activation of CD147, cytokine storm, gut dysbiosis and neuropilin‐1 in SARS‐CoV‐2 infection could be the potential mechanisms for development of AP. Though, it is unclear whether the above‐mentioned factors play a critical role alone or in combination in causing AP in Covid‐19. However, these mechanisms may at least in part explain the possible mechanisms for development of AP in Covid‐19. Further studies are recommended to understand the putative mechanisms of AP in patients with severe SARS‐CoV‐2 infection.

## AUTHOR CONTRIBUTIONS

**Mostafa M. Bahaa:** Conceptualization (equal); data curation (equal); formal analysis (equal); funding acquisition (equal); investigation (equal); methodology (equal); project administration (equal); resources (equal); software (equal); supervision (equal); validation (equal); visualization (equal); writing – original draft (equal); writing – review and editing (equal). **Hayder M. Al‐kuraishy:** Conceptualization (equal); data curation (equal); formal analysis (equal); funding acquisition (equal); investigation (equal); methodology (equal); project administration (equal); resources (equal); software (equal); supervision (equal); validation (equal); visualization (equal); writing – original draft (equal); writing – review and editing (equal). **Ali I. Al‐Gareeb:** Conceptualization (equal); data curation (equal); formal analysis (equal); funding acquisition (equal); investigation (equal); methodology (equal); project administration (equal); resources (equal); software (equal); supervision (equal); validation (equal); visualization (equal); writing – original draft (equal); writing – review and editing (equal). **Athanasios Alexiou:** Conceptualization (equal); data curation (equal); formal analysis (equal); funding acquisition (equal); investigation (equal); methodology (equal); project administration (equal); resources (equal); software (equal); supervision (equal); validation (equal); visualization (equal); writing – original draft (equal); writing – review and editing (equal). **Marios Papadakis:** Conceptualization (equal); data curation (equal); formal analysis (equal); funding acquisition (equal); investigation (equal); methodology (equal); project administration (equal); resources (equal); software (equal); supervision (equal); validation (equal); visualization (equal); writing – original draft (equal); writing – review and editing (equal). **Gaber El‐Saber Batiha:** Conceptualization (equal); data curation (equal); formal analysis (equal); funding acquisition (equal); investigation (equal); methodology (equal); project administration (equal); resources (equal); software (equal); supervision (equal); validation (equal); visualization (equal); writing – original draft (equal); writing – review and editing (equal).

## FUNDING INFORMATION

Nothing to declare.

## CONFLICT OF INTEREST STATEMENT

The authors declare no competing interests.

## Data Availability

All data are available in the manuscript.
